# Global Review of Blue Carbon Ecosystem Microbial Communities

**DOI:** 10.1111/1462-2920.70168

**Published:** 2025-08-16

**Authors:** Christina Birnbaum, Pawel Waryszak, Stacey M. Trevathan‐Tackett, Jennifer L. Bowen, Rod M. Connolly, Carlos M. Duarte, Peter I. Macreadie

**Affiliations:** ^1^ School of Life and Environmental Sciences Deakin University Burwood Victoria Australia; ^2^ School of Agriculture & Environmental Science University of Southern Queensland Toowoomba Queensland Australia; ^3^ Centre for Crop Health University of Southern Queensland Toowoomba Queensland Australia; ^4^ Centre for Sustainable Agricultural Systems University of Southern Queensland Toowoomba Queensland Australia; ^5^ Centre for Nature Positive Solutions, Biology Department, School of Science RMIT University Melbourne Victoria Australia; ^6^ Department of Marine and Environmental Sciences, Marine Science Centre Northeastern University Nahant Massachusetts USA; ^7^ Coastal and Marine Research Centre, Australian Rivers Institute, School of Environment and Science Griffith University Gold Coast Queensland Australia; ^8^ Marine Science Program, Biological and Environmental Science and Engineering Division (BESE) 4700 King Abdullah University of Science and Technology Thuwal Saudi Arabia

**Keywords:** biodiversity, coastal wetlands, global, methanogenesis, microorganisms, nitrogen cycling, sulphate reduction, synthesis

## Abstract

Microbial communities underpin biogeochemical processes in Blue Carbon Ecosystems (BCEs); however, a comprehensive review of geographic patterns in microbial diversity, microbial functions, and distribution is currently lacking. Here, for the first time, we have analysed 70 years (1930–2020) of archaeal, bacterial, and fungal diversity and functions in mangrove, saltmarsh, and seagrass ecosystems to elucidate publication and geographic trends in reporting data in BCEs and to identify knowledge gaps. Of the 649 journal articles analysed, research on BCE microbial communities has focused overwhelmingly on assessing bacterial richness and functions in BCEs. Our gap analysis revealed that only ~25%–50% of the countries that have BCEs have been represented, suggesting that our understanding of archaeal, bacterial, and fungal geographic diversity in BCEs is still incomplete. In the context of taxonomic‐based limitations in our study's approach, we have identified gaps of knowledge in archaeal and fungal sediment biodiversity in saltmarsh and seagrass ecosystems. This significantly impacts our ability to forecast ecosystem services amid current and future human and climate pressures in BCEs. The results from this synthesis could serve as a useful reference for microbial baseline data and research trends in BCEs to develop novel hypothesis‐testing research.

## Graphical abstract

A thorough review of Blue Carbon Ecosystems' global and regional patterns in microbial diversity, functions, and distribution is currently missing. In this study, we have, for the first time, analysed 70 years (1930–2020) of data on archaeal, bacterial, and fungal diversity and reported functions in mangrove, saltmarsh, and seagrass ecosystems. This analysis aims to elucidate temporal and geographic trends in reporting data and patterns in BCEs and to identify existing knowledge gaps.

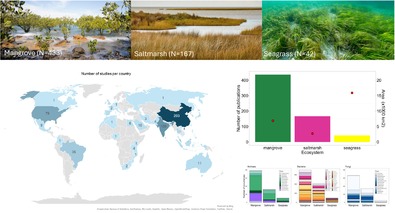

## 1 Introduction

Understanding the biogeochemical processes regulating the movement of micro and macro nutrients is central to controlling the Earth System (Ussiri and Lal 2017). An important thermostat to the Earth System is Blue Carbon Ecosystems (BCEs) a collective term referring to the critically important role of seagrass, saltmarsh and mangrove ecosystems that sequester large quantities of organic carbon. These habitats support net carbon sequestration that is 10–100 times that of terrestrial forests per unit area (Duarte et al. 2013). As BCEs have experienced global declines, the conservation and restoration of BCEs can play a considerable role as carbon sinks in climate change mitigation (Macreadie et al. 2021), while providing a range of ecosystem services. For example, BCEs attenuate coastal erosion (Kazemi et al. 2021), improve water quality (Adame et al. 2015) and serve as biodiversity reservoirs (Farrer et al. 2022).

BCEs are highly heterogeneous environments experiencing considerable temporal and spatial fluctuations in hydrology, salinity, oxygen availability, and climate regimes (Odum 1988). The rhizosphere of BCEs is considered a hotspot of carbon turnover and microbial interactions involving epiphytic and endophytic microbes ranging from bacteria and fungi to viruses and protozoa exchanging nutrients with plant roots and each other (Tarquinio et al. 2019). Plant roots fuel microbial activity by supplying substrate via root exudates and rhizodeposits, as well as providing oxygen in an otherwise oxygen‐depleted environment (Brodersen et al. 2018; Martin et al. 2019). In return, plants receive nutrients needed for growth during microbial soil organic matter (SOM) decomposition (Mozdzer et al. 2016), along with protection from otherwise toxic sulphide.

Soil microbial communities in BCEs account for approximately 30% of the sedimentary carbon (Alongi 2005; Booth et al. 2023). Fungi, bacteria, and archaea play a central role as organic matter (OM) decomposers, thus affecting carbon cycling and storage (Ren et al. 2022, Macreadie et al. 2025). A recent study by Macreadie et al. (2025) reviewed the key factors contributing to the stabilisation and destabilisation of blue carbon, concluding that microbial activity is fundamental to organic carbon mineralisation. Furthermore, mutualistic fungi (i.e., mycorrhizal) and bacteria (i.e., growth promoting rhizobacteria and bacterial endophytes) support plant resistance against abiotic stress by supplying nutrients and growth‐promoting substances (Alfaro‐Espinoza and Ullrich 2015, Daleo et al. 2015, Tarquinio et al. 2019). While BCEs are stressful environments due to low nutrients, low or no oxygen, and high salinity, these conditions have been proposed to facilitate positive plant‐microbe interactions that aid plant establishment and growth (Bertness and Callaway 1994; Lekberg et al. 2018; Valliere et al. 2020). For example, fungi, especially mycorrhizal fungi, and bacteria (growth promoting bacteria and nitrogen fixers) form common mutualistic plant‐microbe associations, whereas archaea and bacteria are responsible for two main metabolic pathways of climatic relevance: methanogenesis and anaerobic methane oxidation (Eme and Doolittle 2015).

Previous reviews have investigated plant‐microbe interactions in aquatic systems (Srivastava et al. 2017) and the ecological importance of plant‐microbe symbioses in coastal ecosystems (Farrer et al. 2022; Crump and Bowen 2023). The global diversity and distribution of root‐ and rhizosphere‐associated fungi in saltmarshes was comprehensively reviewed in Lumibao et al. (2024). The role of the belowground soil microbiome in saltmarsh and mangrove restoration was synthesised in Birnbaum and Trevathan‐Tackett (2023) and biota, including microbial biota, controls on blue carbon in Ren et al. (2022). However, despite accumulating research on BCEs (de Paula et al. 2022) there is a lack of synthesis papers investigating sediment microbial biodiversity and associated functions in BCEs (UNEP 2006; Farrer et al. 2022). Furthermore, little is known about the soil microbial community similarity or dissimilarity across saltmarsh, seagrass, and mangrove ecosystems (Hurtado‐McCormick et al. 2022). However, this is important, as microbial community composition affects critical ecosystem functions, such as decomposition and nutrient cycling in soils (Allison et al. 2013; Reed and Martiny 2013).

Soil microbial communities associated with saltmarsh, seagrass, or mangrove systems may differ due to the differences in vegetation structure (Barreto et al. 2018; Rietl et al. 2016). For example, seagrass ecosystems are dominated by approximately 60 species worldwide belonging to three independent lineages of seagrass (Hydrocharitaceae, Cymodoceaceae complex, and Zosteraceae) (Les et al. 1997). Seagrass rhizospheres harbour both aerobic and anaerobic microorganisms, specifically functionally similar but taxonomically different bacteria, e.g., chemolithotrophic, sulfur‐oxidising, and nitrogen‐fixing bacteria (Ugarelli et al. 2017; Crump et al. 2018; Martin et al. 2018). In saltmarshes, Rietl et al. (2016) found that the sediment bacterial and archaeal, but not fungal communities differed between sites with different plant communities. Similar patterns were reported by Barreto et al. (2018) who found that soil bacterial and archaeal communities were driven by dominant saltmarsh species. However, these differences have not been sufficiently documented due to a lack of studies synthesising the existing knowledge on soil microbial communities across BCEs. Such synthesis would consolidate our understanding of microbial diversity patterns and functional processes in BCEs and identify current research gaps.

Here we provide a review of published literature on soil and rhizosphere microbial communities, including archaeal, bacterial, and fungal communities in the soil or rhizosphere plant communities in BCEs to (a) better understand geographic trends in microbial data, (b) unveil patterns in reported archaeal, bacterial, and fungal diversity, and finally, (c) identify research gaps and future research directions.

## 2 Materials and Methods

**FIGURE 1:**
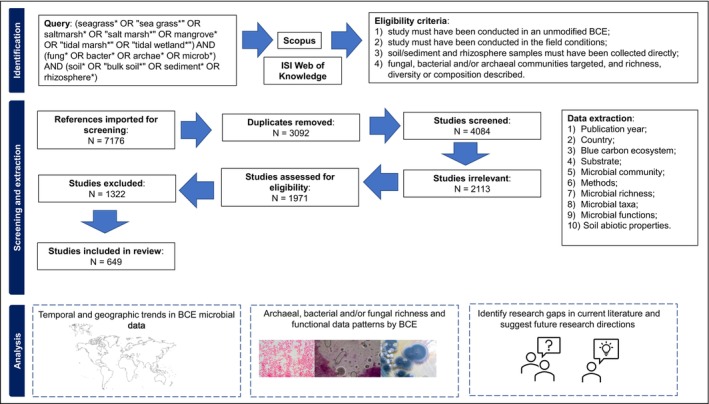
Modified PRISMA flow chart visually summarising the literature screening process. Eligibility criteria and data extraction stated. Analysis summarising the main aims of the systematic review.

The literature searching strategy employed is summarised in Figure 1. We performed a systematic quantitative literature review by searching Scopus (Elsevier, Atlanta, USA) and ISI Web of Knowledge (Core collection; Thomson Reuters, NY, USA) databases on 10 December 2020. These databases were searched through title, abstract, and keywords using the Boolean search string: (seagrass* OR ‘sea grass*’ OR saltmarsh* OR ‘salt marsh*’ OR mangrove* OR ‘tidal marsh*’ OR ‘tidal wetland*’) AND (fung* OR bacter* OR archae* OR microb*) AND (soil* OR ‘bulk soil*’ OR sediment* OR rhizosphere*). Available literature until December 2020 was included.

This search returned 2970 and 3178 papers in Scopus and ISI Web of Knowledge, respectively. All papers were imported into Covidence (Covidence systematic review software, Veritas Health Innovation, Melbourne) for screening. Selection of studies was conducted following PRISMA (Preferred Reporting Items for Systematic Reviews and Meta‐Analyses) protocols (http://www.prisma‐statement.org) which are built‐in into Covidence. Covidence removed duplicates resulting in 4084 papers that were included in title and abstract screening. 2113 studies were classified as irrelevant, and 1971 studies were used for full‐text screening. Following full‐text screening, 1322 studies were excluded because they did not meet the inclusion criteria. A further 19 studies were removed due to incomplete data availability. This resulted in 649 studies that met the following inclusion criteria and were used for data extraction (Figure 1, Table S1 which contains bibliographic information for 649 studies). To assess the microbial communities that are present in unmodified or unmanaged BCEs, we: (1) did not include studies that reported microbial communities from modified, for example, restored or actively managed BCEs; (2) only included studies that were conducted in the field where soil/rhizosphere samples were directly collected; thus studies that reported field manipulative experiments or laboratory experiments were not included, and (3) soil/sediment/rhizosphere samples must have been collected and fungal, bacterial, and/or archaeal communities targeted and richness, diversity, or composition described. We also included studies that assessed microbial functional genes instead of directly targeting and analysing the microbial communities (e.g., *nifH* gene for nitrogen fixation). The following 10 data attributes were extracted from 649 studies: (a) year of publication, (b) country where research was conducted, (c) BCE where research was conducted, that is, seagrass, saltmarsh, or mangrove, (d) substrate from which microbial communities were analysed (i.e., soil/sediment, which were treated as the same substrate (henceforth soil), and rhizosphere), (e) microbial communities described (i.e., bacteria, fungi or archaea), (f) methods used to analyse microbial communities (e.g., culture‐dependent, sequencing), (g) reported microbial richness, (h) reported microbial taxa, (i) reported microbial functions (either putative or directly assessed by authors) and (j) soil abiotic properties assessed. Although we only targeted studies with a focus on soil or rhizosphere microbial communities, when host plants were mentioned, these data were extracted too. Data were analysed and plotted using R (R Core Team 2020) and Microsoft Excel 365 (Version 2208, Microsoft Corporation). Formal gap analysis was conducted by assessing the number of countries where the BCE data were reported and compared to the reported country results from this review. Then the area of BCEs was analysed against the number of publications reviewed in this study. The country and area data for BCEs was retrieved from Bertram et al. (2021). Only the abundant (> 1%) phyla were extracted from papers; thus the phyla data should be interpreted with caution as it do not reflect all phyla reported in papers. Furthermore, microbial taxonomy is prone to frequent amendments to phyla names, and thus, when analysing papers from different decades, this needs to be considered. Here, we cleaned all phyla data by cross‐checking with the latest taxonomic assignments *sensu* Ludwig et al. (2021).

Venn diagrams for unique (i.e., only reported from either seagrass, saltmarsh or mangrove ecosystem) and shared phyla between BCEs were built using the most abundant (usually > 1%) phyla reported directly in studies (40% of studies). The remaining phyla were extrapolated from higher taxonomic levels reported in 50% of studies. Approximately 10% of studies did not report any information on the taxonomy of microbial groups studied. Putative functions of archaea, bacteria, and fungi that were reported in reviewed studies were recorded and merged with reported phyla information provided in those studies for analysis.

## 3 Results

### 3.1 Publication Trends in Belowground BCE Microbial Studies

**FIGURE 2:**
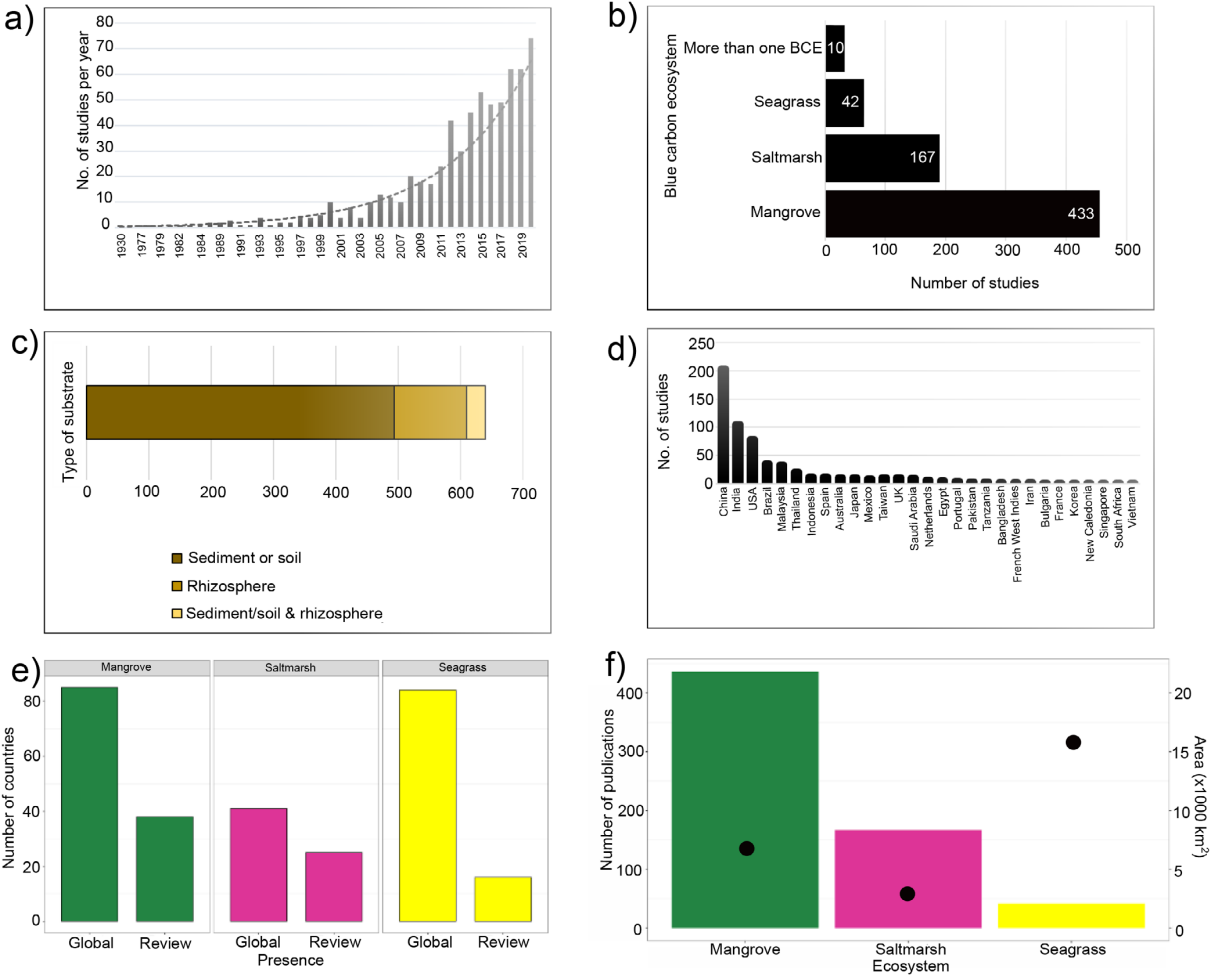
Number of total studies (a) published from 1930 to 2020 on soil microbial communities in blue carbon ecosystems; (b) number of studies by ecosystem type, “Other” refers to seven studies analysed soil microbial communities in more than one BCE; (c) substrate studied where soil microbial communities were analysed from; (d) number of studies by country; (e) Gap analysis showing the total number of countries where mangroves, saltmarshes, and seagrasses occur *sensu* Bertram et al. (2021) and the number of countries per ecosystem that were included in the 649 studies in this review; (f) Gap analysis showing number of publications per ecosystem as reviewed across 649 studies and the total area (km^2^) of each ecosystem (indicated by the red point) *sensu* Bertram et al. (2021).

The studies included in our review were published between 1930 and 2020 (Figure 2a). The first study published in 1930 described the saltmarsh soil fungal communities from Wales (Elliott 1930). The total mean number of studies published on soil microbial communities in BCEs between 1930 and 2020 was on average 15 studies per year (Figure 2a). From 2000 to 2020, the mean number of studies published per year doubled to almost 30, and in 2020 the total number of studies published was 74 (Figure 2a).

Soil microbial communities were studied predominantly in mangroves (66% of studies), of which 36% (157 studies) were focused solely on taxonomic description of soil microbial communities (Figure 2b). These studies were analysed separately from the rest of the 276 mangrove studies (see below). Approximately 25% of studies analysed soil microbial communities from saltmarshes, followed by only 6% in seagrass ecosystems (Figure 2b). Soil microbial communities were studied predominantly from the soil (77% of studies) followed by the rhizosphere (18% of studies) (Figure 2c).

A third of all studies were from China (205), followed by India (105), USA (79), Brazil (36) and Malaysia (34) (Figure 2d). In China and India, 70% and 95% of studies, respectively, were on mangrove soil microbial communities. In China, 25% of studies were on saltmarsh, and 5% on seagrass soil microbial communities. In India, only three studies were on saltmarsh and one study on seagrass and tidal wetland soil microbial communities. Whereas in the USA, 86% of studies were on saltmarsh soil microbial communities, followed by six studies on seagrass and four studies on mangrove soil microbial communities. In Brazil and Malaysia, close to 100% of all studies were on mangrove soil microbial communities.

Soil physical and chemical properties were reported in 74% of studies. A total of 86 different soil properties were reported, of which 19 were reported from more than 1% of studies (Table 1). The three dominant soil attributes were pH, soil salinity, and organic carbon (Table 1).

**TABLE 1:** Number of studies and their proportion of total studies (%) for reported 19 soil physical or chemical properties from 649 studies analysed in this review. Only soil properties that were assessed in < 1% of studies are shown.

Soil/sediment property	No. of studies	Proportion of total studies (%)
pH	83	12.4
Sediment salinity	65	9.7
Organic carbon	40	6
Sediment temperature	32	4.8
Total nitrogen	32	4.8
Soil ammonium	31	4.6
Organic matter content	28	4.2
Dissovled nitrate	28	4.2
Electrical conductivity	19	2.8
Soil moisture	18	2.7
Granulometry	15	2.2
Redox potential	14	2.1
Dissolved nitrate	14	2.1
Total phosphorus	11	1.6
Total carbon	11	1.6
Dissolved iron	10	1.5
Dissolved phosphorus	9	1.3
Soil texture	9	1.3
Magnesium	8	1.2

### 3.2 Microbial Diversity and Functions Across BCEs

#### 3.2.1 Mangrove Microbial Communities

**FIGURE 3:**
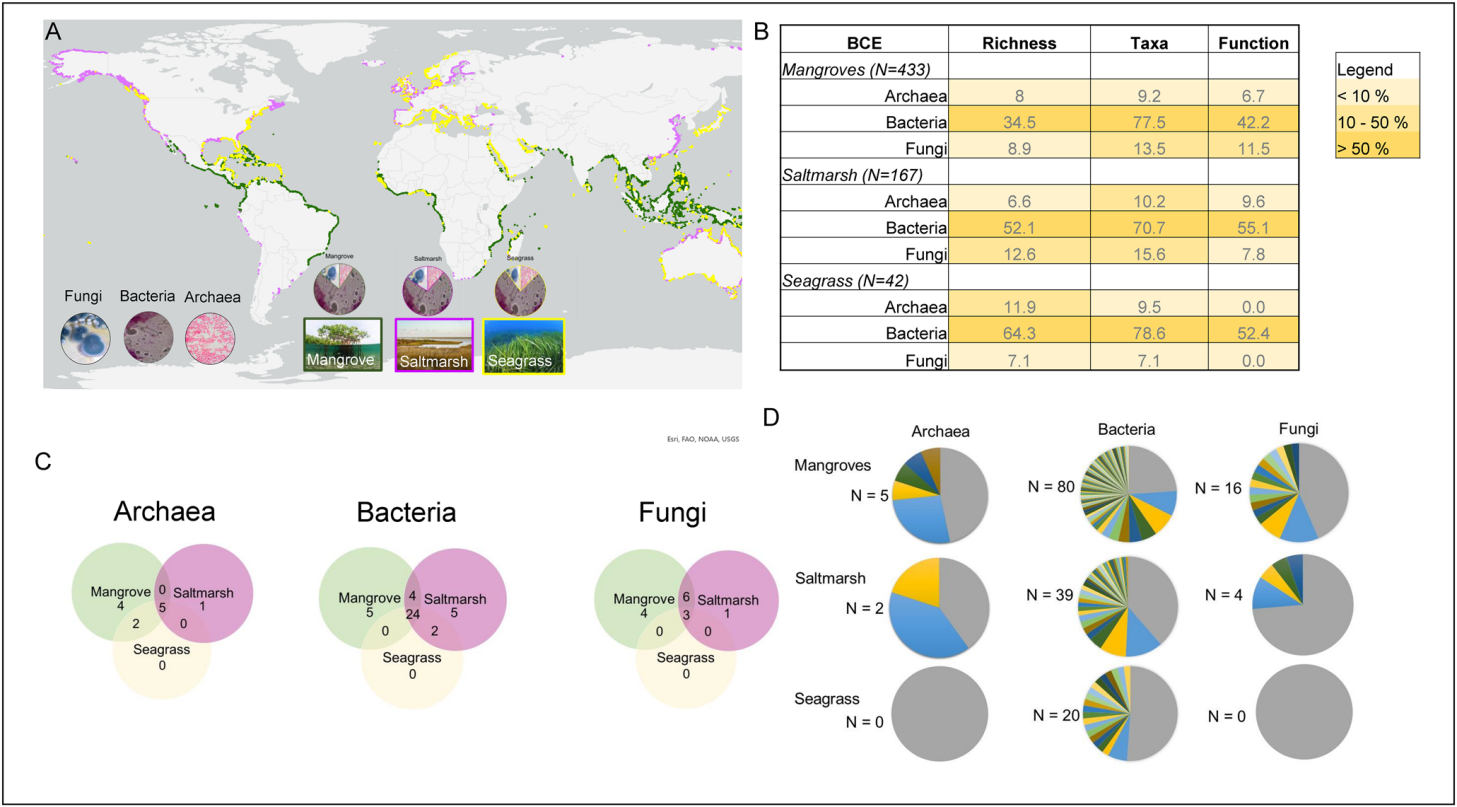
(a) Global distribution of saltmarsh (purple), seagrass (yellow) and mangrove (green) ecosystems and proportion of studies that reported archaea, bacteria or fungi across BCE in this synthesis; (b) proportion of studies reporting archaeal, bacterial or fungal richness, microbial taxonomic description and microbial functions across BCEs from studies reviewed in this synthesis, *N* is the total number of studies per BCE; (c) Venn diagrams showing unique and shared phyla for archaea, bacteria or fungi across BCEs and (d) proportion of microbial functions described for studies that focused on archaea, bacteria or fungi, *N* is the number of different unique functions described per microbial group per ecosystem, grey area refers to proportion of studies that did not state any microbial functions. Full list of all functions described in studies reviewed here is available in the Table S2.

**FIGURE 4:**
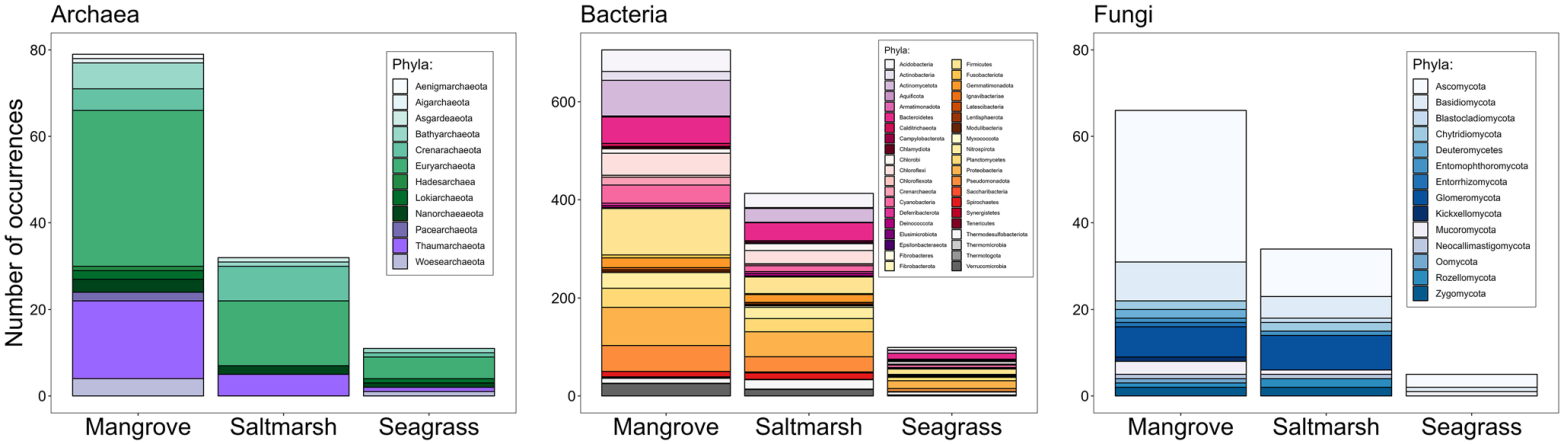
Number of occurrences for most abundant phyla (> 1%) of archaea, bacteria and fungi across BCEs as reported in the studies reviewed in this synthesis, note different *y*‐axis on sub‐panels.

**FIGURE 5:**
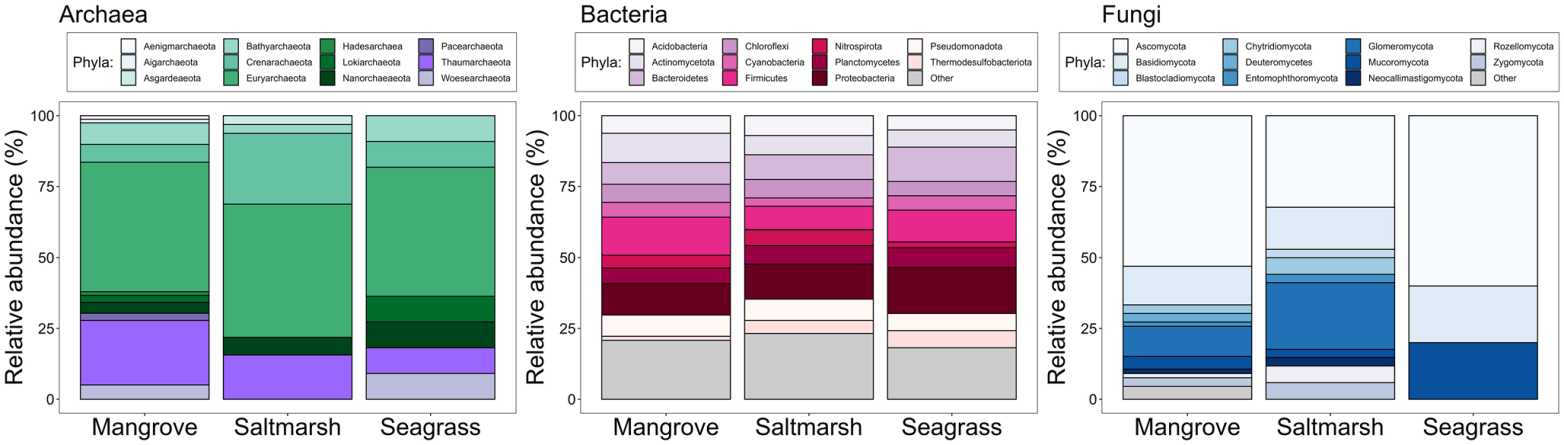
Relative abundance of 12 archaeal phyla, 11 most abundant bacterial and fungal phyla across mangrove, saltmarsh and seagrass ecosystems as reported in this review. “Other” refers to all remaining phyla in these groups. For the full list of all phyla please see Tables S4.

**FIGURE 6:**
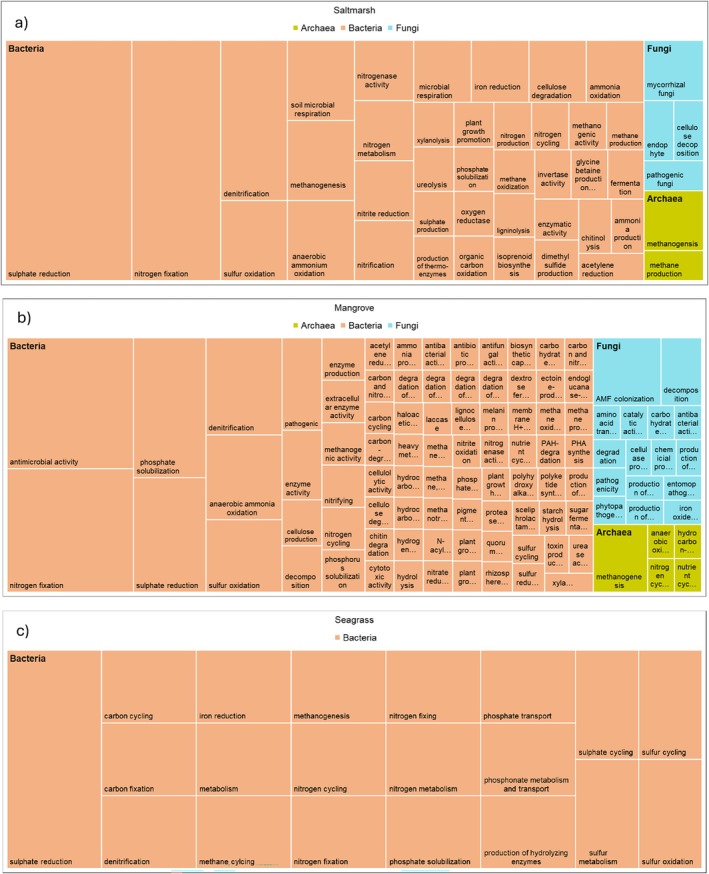
TreeMap of putative functional diversity of archaea, bacteria and fungi across BCEs as reported in the studies reviewed in this synthesis. Rectangles are proportionally sized according to the amount of data in each category. Full list of putative functions is available in Table S2.

Excluding the taxonomic studies, 78% of studies on mangrove soil microbial communities were from China (82), India (78), Brazil (32), Malaysia (15) and Mexico (8). In mangrove ecosystems, 41% of studies (178) reported on soil bacterial communities, followed by fungi 9% (39 studies) and 5% (22) of studies that reported on both archaea and bacteria. Furthermore, bacterial taxa were described in 77.5% of studies, bacterial richness in 34.5% of studies, and bacterial functional attributes were assessed in 42.2% of studies (Figure 3b). Thirty‐three bacterial phyla were described from mangrove studies, which were collectively listed 706 times across 433 mangrove studies (note, one phylum could be listed multiple times in a single study) (Figure 4). Of those, Firmicutes (94 studies), Proteobacteria (78), Actinomycetota (73), Bacteroidetes (54), and Pseudomonadota (53) were mentioned in 50% of the studies (Figures 4 and 5). Five bacterial phyla were only reported from mangroves, that is, Armatimonadota, Elusimicrobiota, Fibrobacteres, Synergistetes, and Thermomicrobia (Figure 4). Eighty unique bacterial functions were reported (Figures 3d and 6a and Table S2). For example, 18 studies reported antimicrobial activity, 17 nitrogen fixation, and 11 phosphate solubilisation (Figure 6a, Table S2). Sixty‐three bacterial functions were only reported once (Figure 3d and Table S2).

Fungal richness, taxonomy, and functions were described in 8.9%, 13.5% and 11.5% mangrove studies, respectively (Figures 3b and 6a). A total of 13 phyla were described, with Ascomycota (35), Basidiomycota (9), Glomeromycota (7), Mucoromycota (3) and Chytridiomycota (2) reported collectively 85% of the time (Figure 5). Four fungal phyla were unique to mangroves, that is, Deuteromycetes, Entorrhizomycota, Kickxellomycota and Oomycota (Figure 3c). Out of 39 studies that assessed only soil fungal communities, 22 studies reported fungal functions. For example, arbuscular mycorrhizal colonisation was reported in five studies (da Silva et al. 2017; Ramirez‐Viga, Guadarrama, et al. 2020; Ramirez‐Viga, Ramos‐Zapata, et al. 2020; Kumar and Ghose 2008; Wang et al. 2015), while fungal decomposition was reported in three (Ulken et al. 1990; Latiffah et al. 2010; and Nazim et al. 2013).

Archaeal richness, taxonomy, and functions were reported in less than 10% of studies (Figures 3b and 6a and Table S2). Eleven unique archaeal phyla were reported from mangrove studies (Figures 3c and 6a). Euryarchaeota (36 studies) and Thaumarchaeota (18 studies) were the dominant phyla reported from mangroves (Figures 3c and 5). Four archaeal phyla were unique to mangroves, that is, Aenigmarchaeota, Aigarchaeota, Hadesarchaea, and Pacearchaeota (Figures 3, 4, 5). Out of 15 studies that reported on archaeal communities, four studies reported on methanogenesis, two on nutrient cycling, and six studies did not describe any functional attributes of archaea (Figure 3d, Table S2).

Host plants associated with microbial communities in mangrove ecosystems were reported in 51% of studies, with a total plant host richness of 38 species from 20 mangrove and saltmarsh genera (*Avicennia, Rhizosphora, Buguiera, Sonneratia, Ceriops, Conocarpus, Kandelia, Sueada, Acanthus, Acrostichum, Aegiceras, Excoecaria, Laguncularia, Lumnitzera, Nypa, Porteresia, Salicornia, Sesuvium, Spartina, Xylocarpus*).

There were 157 (36%) studies that focused entirely on single species taxonomic descriptions. Of these, 145 studies focused on bacterial and 2 on archaeal ( *Methanosarcina semesiae* sp. nov. (Lyimo et al. 2000); Thorarchaeota (Liu et al. 2018)) taxonomic descriptions using 16S rRNA sequencing methods. The remaining five studies described fungal species (*Aspergillus* sp. (Tao et al. 2018); *Vanakripa rhizophorae*, *Polyschema nigroseptatum* (Mota et al. 2008); *Candida sanitii* sp. *nov., Candida sekii sp.nov., Candida suwanaritii* (Limtong et al. 2008); *Candida saraburiensis* sp. *nov., Candida prachuapensis* sp. *nov*. (Nitiyon et al. 2011)). Eighty‐one different bacterial genera were described, with 15% of bacterial studies describing species from the *Streptomyces* genus, 6.4% species from the *Micromonospora* genus and 3.2% species from the *Agromyces* genus. 45% of studies focusing exclusively on single species taxonomic descriptions originated from China, followed by India (13%), Malaysia (12%), Thailand (11%) and Japan (5%).

#### 3.2.2 Saltmarsh Microbial Communities

Nearly half of the studies (41%) describing microbial communities from saltmarshes were from the USA, followed by China (28%) and Spain (6%). In saltmarsh communities, bacterial richness and functions were described in > 50% of studies, fungal richness was described in 12.6% of studies, and functional properties in < 10% of studies (Figure 3b). Archaeal richness and functional properties were described the least, in < 10% of studies (Figure 3b). Six archaeal phyla were reported from saltmarsh studies, that is, Euryarchaeota, Crenarchaeota, Thaumarchaeota, Nanorchaeaeota, Bathyarchaeota, and Asgardeaeota, of which one phylum was unique to only saltmarshes, that is, Asgardeaeota (Figures 4 and 5).

Thirty‐five bacterial phyla were reported from the saltmarsh studies (Figure 4). Of these, Proteobacteria, Bacteroidetes, Firmicutes, Pseudomonadota, Acidobacteria, Actinomycetota, Chloroflexi, Planctomycetes, and Nitrospirota were mentioned in more than 20 studies (i.e., occurrences) (Figures 4 and 5). There were five bacterial phyla unique to saltmarshes, that is, Aquificota, Fibrobacterota, Modulibacteria, Saccharibacteria, and Thermotogota (Figures 3c and 4).

Ten fungal phyla were reported from saltmarsh studies: Ascomycota, Glomeromycota, Basidiomycota, Chytridiomycota, Rozellomycota, Zygomycota, Blastocladiomycota, Entomophthoromycota, Mucoromycota, and Neocallimastigomycota (Figures 4 and 5). One fungal phylum was unique to saltmarshes, that is, Blastocladiomycota (Figure 3c). In saltmarshes, microbial functions were reported most for bacteria and considerably less for archaea and fungi (Figures 3d and 6b). For example, two studies focusing on archaea reported methanogenesis (Table S2). Studies that described fungi reported mycorrhizal and endophyte status (*n* = 3), cellulose decomposition (*n* = 1) and pathogenic fungi (*n* = 1) (Figure 3d, Table S2). Bacterial functional diversity in saltmarshes was described in 55% of studies (Figure 3b) with 17 studies reporting sulphate reduction, 12 studies nitrogen fixation, and 6 studies denitrification (Figure 6b, Table S2).

Host plants associated with microbial communities in saltmarsh ecosystems were reported in 31% of studies with a total plant host richness of 38 species. Of these studies, 19% reported *Spartina alterniflora* , followed by *Phragmites australis* (4%), *Juncus roemarinus* (2%) and *Cyperus malaccensis* (1.8%).

#### 3.2.3 Seagrass Microbial Communities

A considerable proportion of seagrass studies were reported from China (10), USA (6), Australia (5), France (4), Portugal (3) and Saudi Arabia (3). In seagrass ecosystems, 9.5% of studies reported on soil fungal communities, 11.9% on archaeal, and 73.8% on bacterial communities (Figure 3a). Three fungal (Ascomycota, Basidiomycota and Mucoromycota), seven archaeal (Bathyarchaeota, Crenarachaeota, Euryarchaeota, Lokiarchaeota, Nanorchaeaeota, Thaumarchaeota and Woesearchaeota) and 26 bacterial phyla were reported from the seagrass ecosystems (Figures 4 and 5). Bacterial community richness and function were reported in > 50% of studies (Figures 3b and 6c and Table S2), whereas for both archaea and fungi, richness was reported in ~10% of studies (Figure 3b) and microbial functions were not reported for archaea nor fungi (Figures 3b,d and 6c, Table S2). For bacteria, several functions were assessed involved in carbon (e.g., methane cycling, nitrogen (e.g., denitrification), nitrogen fixation), sulfur (e.g., sulfate reduction) and phosphorus (e.g., phosphate solubilisation) biogeochemical cycles (Figure 3d and Table S2). Host plants associated with microbial communities in seagrass ecosystems were reported in 28% of studies, with a total plant host richness of 13 species (*Cymodoceae nodosa, Enhalus acoroides, Halodule pinifolia, H. uninervis, Halophila ovalis, H. pinifolia, H. stipulacea, Posidonia oceanica, Thalassia hemprichii, T. testudinum, Zostera marina, Z. noltii, Z. japonica, Z. marina*).

### 3.3 Shared Microbial Communities Across BCEs

Mangroves shared the highest number, that is, 24 bacterial phyla (Acidobacteria, Actinobacteria, Actinomycetota, Bacteroidetes, Campylobacterota, Chlamydiota, Chlorobi, Chloroflexi, Chloroflexota, Crenarchaeota, Cyanobacteria, Deferribacterota, Deinococcota, Firmicutes, Fusobacteriota, Latescibacteria, Lentisphaerota, Nitrospirota, Planctomycetes, Proteobacteria, Pseudomonadota, Spirochaetes, Thermodesulfobacteriota, Verrucomicrobia) with saltmarsh and seagrass ecosystems (Figure 3c). There were four bacterial phyla that were shared between mangroves and saltmarsh, that is, Calditrichaeota, Gemmatimonadota, Ignavibacteriae, and Tenericutes (Figures 3c and 5). There were two bacterial phyla shared between saltmarsh and seagrass ecosystems, that is, Epsilonbacteraeota (formerly within Proteobacteria, reclassified as its own phylum in 2017 (Waite et al. 2018)), and Myxococcota (Figures 3c and 4).

Three fungal phyla were shared among all three ecosystems, that is, Ascomycota, Basidiomycota, Mucoromycota (Figures 3c and 5). Mangroves shared the highest number of fungal phyla with saltmarsh ecosystems, that is, Chytridiomycota, Entomophthoromycota, Glomeromycota, Neocallimastigomycota, Rozellomycota, and Zygomycota (Figures 3c and 4). There were no shared fungal phyla between mangroves and seagrass ecosystems (Figure 3c). There were no unique shared fungal phyla between mangroves and saltmarsh ecosystems nor between saltmarsh and seagrass ecosystems (Figure 3c).

Five archaeal phyla were shared among all three ecosystems, that is, Bathyarchaeota, Crenarachaeota, Euryarchaeota, Nanorchaeaeota and Thaumarchaeota (Figures 3c and 5). Mangroves shared the highest number of archaeal phyla with seagrass ecosystems, that is, Lokiarchaeota and Woesearchaeota (Figures 3, 4, 5). There were no unique archaeal phyla shared between saltmarsh and mangrove, and saltmarsh and seagrass ecosystems (Figure 3c).

There were no unique archaeal, bacterial, or fungal phyla in seagrass ecosystems, with all phyla shared with saltmarsh and mangrove ecosystems (Figures 3c and 4).

### 3.4 Associations Between Putative Functions and Archaeal, Bacterial, and Fungal Phyla Across All BCEs

For archaeal communities, 16 studies reported both phyla and the putative functions of members belonging to these phyla. These putative functions were anaerobic oxidation of methane (Crenarchaeota, Euryarchaeota, Thaumarchaeota), hydrocarbon degradation (Euryarchaeota, Thaumarchaeota), methanogenesis (Crenarchaeota, Euryarchaeota, Thaumarchaeota), nitrogen cycling (Euryarchaeota, Thaumarchaeota) and nutrient cycling (Euryarchaeota, Thaumarchaeota) (Table S3).

For bacterial communities, 450 studies reported both phyla and the putative functions of members belonging to these phyla. Of these, 82 unique putative functions were attributed to members of specific phyla (Table S3). For example, the 10 most common putative bacterial functions were enzyme activity (members from 21 bacterial phyla), nitrogen fixation (members from 16 bacterial phyla), phosphorus solubilisation (members from 21 bacterial phyla), sulfate reduction (members from 10 bacterial phyla), phosphate solubilisation (members from 7 bacterial phyla), antimicrobial activity (members from 10 bacterial phyla), sulfur oxidation (members from 17 bacterial phyla), quorum sensing (members from 14 bacterial phyla), decomposition (members from 12 bacterial phyla) and carbon cycling (members from 11 bacterial phyla). For all listed bacterial putative functions and associated phyla, please see Table S3.

For fungal communities, 22 studies reported 13 unique putative functions and their associated phyla (Table S2). For example, arbuscular mycorrhizal colonisation was reported only from members of Glomeromycota; decomposition was reported for Ascomycota, Chytridomycota and Mucoromycota (Table S3).

### 3.5 Methods Used to Analyse Microbial Communities

Our results showed that HTS sequencing was the most reported assessment technique of archaeal, bacterial, or fungal communities across mangrove (69% of studies), saltmarsh (56% of studies) and seagrass (66% of studies) ecosystems (Figure S1). This was followed by culture‐dependent methods, with 18% of mangrove, 15% of saltmarsh, and 12% of seagrass studies reported using these methods (Figure S1).

## 4 Discussion

Microbial communities are critical in BCEs because of the important role they play in microbial food webs, primary production, detritus degradation, and nutrient cycling and carbon sequestration (Crump and Bowen 2023). Furthermore, microbial communities that associate with seagrasses, mangrove, and saltmarsh plant communities are critical for host physiology, host‐pathogen interactions, plant growth promotion, and toxin removal (Crump and Bowen 2023). Plant‐microbe interactions in BCEs are directly influenced by the environmental conditions as well as the microbial pool in the soil and the rhizosphere. Thus, it is imperative to understand soil and rhizosphere microbial diversity and distribution patterns to better predict their plant‐microbial dynamics, response to future environmental change, and role in BCE ecosystem services and restoration.

Here, we have comprehensively synthesised 70 years' worth of data on global microbial diversity in BCEs to elucidate publication trends in microbial data, assess patterns in reported global archaeal, bacterial, and fungal diversity, and finally, to identify research gaps in current literature and suggest future research directions.

### 4.1 Publication Trends in BCE Microbial Data

From the 1930s to 2007, there were on average 10 studies published on microbial communities in BCEs. However, since 2013, there has been a three‐fold increase in journal articles published on microbial communities in BCEs, with an average of 35 journal articles published per year to more than 75 articles published in 2020. Compared to the total number of studies published on BCEs (~350 per year) in 2020 as reviewed by Duarte and Macreadie (2022), studies that have assessed soil microbial communities constitute 20% of all articles published in that year. The increase in the number of studies focusing on assessing soil or rhizosphere microbial diversity is likely caused by several factors: (1) the greater appreciation of the importance the soil microbial communities play in regulating ecosystem functions and services in BCEs (Crump and Bowen 2023); (2) overall scientific journal article output has grown exponentially since 2000 with the rise in open‐access publishing, expansion of digital databases like Scopus and Web of Science, and growth in research output from countries like China and India (Thelwall and Sud 2022). Since 2021, 1498 new articles have been published in Scopus as of July 2025 that match our key word search string indicating that this is a rapidly growing research field. Furthermore, the onset of high throughput sequencing (HTS) tools has greatly contributed to our ability to assess the diversity and genomic functions of microbes in BCEs (Crump and Bowen 2023). Some authors have, however, identified persistent technical, methodological, and theoretical challenges that prevent a synthesis of multiple studies with currently available microbial HTS data from BCEs, calling for more unified and comparable methodologies (Hurtado‐McCormick et al. 2022).

Studies from China focusing on mangrove bacterial communities constitute most studies reviewed here. Despite this, our gap analysis revealed that we have only scratched the surface in describing the microbial communities across BCEs (Trevathan‐Tackett et al. 2019; Farrer et al. 2022). For example, seagrass microbial communities have been described the least, that is, from 16 countries, despite seagrasses occurring in 84 countries globally and occupying the highest surface area compared to mangrove and saltmarsh communities. This may be due to several factors, for example, lack of access to survey remote seagrass beds, lack of new seagrass researchers trained in identifying seagrass families, and lack of national and international approaches to seagrass research at different jurisdiction levels (York et al. 2017).

External abiotic factors that shape microbial diversity and functions were also reviewed, with pH, salinity, organic carbon, sediment temperature, and total nitrogen reported in 38% of the studies (Table 1). These environmental factors play significant roles in influencing blue carbon stabilisation and destabilisation (Macreadie et al. 2025). For example, the decline in pH related to acidification may be the main driver of methanogenesis, which contributes to stable carbon release into the atmosphere if methanotrophs are absent (Chuan et al. 2020). Conversely, an increase in pH in BCEs has been reported to rapidly solubilise organic matter and alter microbial community structure (Anderson et al. 2018). Salinity can act as a critical environmental filter for microbial communities, with consequences for microbial diversity and associated biogeochemical processes still poorly understood (Zhang et al. 2021). For instance, Wang et al. (2018) found that salinity elevation decreased the activity and abundance of denitrifiers in mangrove sediment; Ikenaga et al. (2010) found evidence for salinity shaping the bacterial communities, with higher prevalence of *Chloroflexi* in mangroves and *Proteobacteria, Spirochetes, and Planctomycetes* dominating the seagrass ecosystems. Sediment temperature is becoming increasingly relevant in the context of global climate change and will influence several biogeochemical processes in BCEs (Gruber 2011). Thus, it is expected that these environmental factors have been measured in almost half of all studies reviewed here.

### 4.2 Patterns in Global Archaeal, Bacterial and Fungal Diversity and Functions in BCEs

It is becoming increasingly evident that soil microbial communities play critical roles across space and time in supporting ecosystem functions, ecosystem resilience, and restoration success in BCEs (Escalas et al. 2019; Birnbaum and Trevathan‐Tackett 2023). Better understanding both the microbial biodiversity, as well as the diversity of functions microbial communities perform, has the potential to elucidate how to best manage, conserve, and restore BCEs. In fact, the term functional diversity has been recognised as the missing link between biodiversity patterns and ecosystem functions in terrestrial ecosystems but is increasingly recognised as a central driver of ecosystem services (Bardgett and van der Putten 2014; Carrara et al. 2015). Here, we reviewed both the richness of microbial species as well as their reported functional diversity across BCEs.

Research attention was highest for bacterial communities across saltmarsh, seagrass, and mangrove ecosystems, with approximately 70% of studies reported assessing bacterial communities. Bacterial richness, taxonomic, and functional diversity were reported considerably more often, that is, from 42% to 78% of studies, than for fungal or archaeal communities. Specifically, 80 different bacterial functions were reported from mangrove studies, 39 from saltmarsh studies, and 20 from seagrass studies. This result is not surprising, as bacteria are the primary decomposers of organic matter, performing diverse metabolic functions that control the biological availability of nitrogen, phosphorus, sulfur, iron, manganese, and silicon (Crump and Bowen 2023). Bacteria, along with other BCE microbes, play an important role in carbon stabilisation/destabilisation in BCEs via decomposing carbon from the detritus and sediments (Macreadie et al. 2025). For example, aerobic and anaerobic respiration may contribute to blue carbon destabilisation, while denitrification, sulphate, and iron reduction contribute to the stabilisation of blue carbon (Macreadie et al. 2025). Additionally, bacteria also serve as an important food source for protists in microbial food webs (Crump and Bowen 2023). A total of 40 different bacterial phyla were reported across BCEs as most abundant, with 24 (~50%) phyla overlapping between the three BCEs, suggesting high similarity in bacterial taxa across the BCEs. Many of these phyla are known to be involved in pivotal ecosystem processes in BCEs, for example, methane and sulfur cycles, as reviewed in detail in Crump and Bowen (2023). Notably, we did not find bacterial phyla unique to only seagrass ecosystems, suggesting that seagrass ecosystems' bacterial diversity is similar to that of saltmarsh and mangroves. Similarities in overlapping phyla across mangrove, saltmarsh, and seagrass ecosystems are not surprising, as these ecosystems experience similar environmental filtering (Kraft et al. 2015). For example, salinity is a key driver of global environmental microbiome community structure that selects for specific microbial taxa (Lozupone and Knight 2007). Additionally, many of these taxa commonly found in these ecosystems are adapted to the anoxic sediments of all BCE systems. For instance, many of these microbial taxa are either obligate or facultative anaerobes that have metabolisms that still function in the absence of oxygen, albeit less efficiently, and this decrease in efficiency of anaerobic metabolism is one reason that carbon storage is high in BCEs, that is, due to slower decomposition under anaerobic conditions as compared to aerobic terrestrial conditions.

Although archaea are responsible for methanogenesis and are often assessed in concert with bacteria, constituting prokaryotes, their richness, taxonomy and functional diversity were rarely the sole focus of studies reviewed here (i.e., 0%–10% that assessed richness, taxa or function). Archaeal richness and taxa were most reported from seagrass studies, although no functions were reported from seagrass studies and only few from saltmarsh (2) and mangrove (5) ecosystems. The lack of archaeal representation may be due to biases in prokaryotic primer selection (e.g., Bahram et al. 2019, McNichol et al. 2021), resulting in low relative abundances of archaea at the phyla level in this study. Recent studies, however, suggest that the archaeal community or the ‘archaeome’ is now increasingly recognised as an important component of host‐associated microbiomes in protists and plants, where they contribute to methane production, ammonium oxidation, and disease‐relevant processes (Borrel et al. 2020). This clearly warrants future research, which may be facilitated by the optimisation and use of archaeal‐specific primers in marine environments (Bahram et al. 2019; Gantner et al. 2011).

We found that fungal communities were reported at similar low frequencies as archaeal communities. Fungal distribution in coastal wetlands is likely to be more dependent on the plant communities present, as fungi, for example, arbuscular mycorrhizal fungi, are closely associated with plants (Wang et al. 2022). Fungi play a critical role in coastal ecosystems, being the primary decomposers of standing dead biomass in saltmarshes (Buchan et al. 2003) and facilitating saltmarsh denitrification (Kearns et al. 2019). However, historically, there has been less attention on fungal communities relative to bacteria, as in anoxic wetland conditions, many ecosystem processes were thought to be driven predominantly by prokaryotes (Khan 2004). Thus, compared to bacterial communities, studies on fungal community diversity, function, and distribution in BCEs have been lagging (Lumibao et al. 2024). Fungal richness, taxonomy, and functions' reporting was skewed towards studies reporting on saltmarshes. We found 14 abundant fungal phyla reported, with Ascomycota, Glomeromycota, and Basidiomycota reported most often from saltmarshes. These findings are supported by other studies that have found that soil fungi, rather than bacteria, are more critical in saltmarshes, contributing to higher soil multifunctionality (i.e., nine functions related to C, N and P cycling) (Li et al. 2022; Kearns et al. 2019). We also found that there was higher overlap in fungal phyla between mangroves and saltmarshes than between mangroves or saltmarsh and seagrass, suggesting that seagrass ecosystem fungal communities have low diversity at the phylum level. In contrast, fungal functional diversity was reported most from mangroves, amounting to 16 unique functions and four unique functions from saltmarshes. Taken together, and despite their functional importance in supporting plant growth and decomposition, we know surprisingly little about soil fungal communities in BCEs.

### 4.3 Study Limitations

Systematic review is a useful method to synthesise published literature to identify patterns in data collection, sampling, and analyses. It is an especially valuable exercise to summarise published knowledge and identify research gaps to inform future directions. However, as with all methods, there are limitations (Haddaway et al. 2020). For example, a common challenge with reviews involves the type of databases that are used, usually Scopus and ISI Web of Science, and the omission of grey literature and traditional knowledge (Franceschini et al. 2016). By using the PRISMA approach, we avoided common challenges associated with systematic reviews (Haddaway et al. 2020). However, some challenges remained inherent to how data was reported in reviewed papers and our capacity to meaningfully synthesise it. For example, only the abundant (> 1%) phyla were extracted from papers; thus, the phyla data should be interpreted with caution as it does not reflect all phyla reported in papers. Furthermore, microbial taxonomy is prone to frequent amendments to phyla names, and thus, when analysing papers from different decades, this needs to be considered. Here, we cleaned all phyla data by cross‐checking with the latest taxonomic assignments *sensu* Ludwig et al. (2021). We also acknowledge that the putative microbial functions and their association to reported phyla should be interpreted with caution as not all members of a phylum would perform the assessed function. Functional traits frequently do not align neatly with broad taxonomic categories, necessitating more detailed taxonomic distinctions, such as genus or species level, for processes like nitrogen fixation, methanotrophy, and nitrification, which were beyond the scope of this manuscript. Finally, specific microbial functions, for example, sulphate reduction, may have been underreported here because old nomenclature placed them in Proteobacteria (Deltaptroteobacteria).

### 4.4 Key Knowledge Gaps and Future Research Recommendations

Here, we have reviewed the global patterns in the archaeal, bacterial, and fungal communities in soil and the rhizosphere of mangroves, saltmarsh, and seagrass communities. Based on the results from this review, we identified several knowledge gaps that warrant future research:

1. More research is needed to address the geographic bias found in reporting on microbial communities in BCEs. Compared to the global distribution of mangroves, saltmarsh, and seagrasses, microbial communities have been assessed in ~50% of countries where mangroves and saltmarshes have been reported and ~25% of countries where seagrass microbial communities have been reported.
2. Archaeal and fungal soil communities in BCEs are severely underreported, especially in seagrass ecosystems, and we lack a basic understanding of their species richness, species diversity, and functional diversity. This is important as archaea and fungi play critical roles in driving the ecosystem processes in BCEs related to carbon retention and recalcitrance. This has implications for assessing carbon cycling in BCEs, BCEs potential to sequester carbon, and our ability to conserve or restore BCEs (Macreadie et al. 2025). Furthermore, fungi form close associations with plants, and better understanding their presence or absence in BCEs has important ecological implications for the host‐microbiome relationship, which can contribute to our ability to better conserve or restore these systems.
3. Coastal soils contain many important microorganism groups; we recommend that future studies focus on better understanding the synergies between archaea, bacteria, and fungi, which should be inventoried in BCEs to provide a more holistic understanding of microbial complexities in BCE soils (Crump and Bowen 2023). Our review could be used as a starting point for mapping specific microbial taxa at a genus level to their (putative) function in BCEs. Here, we used broader taxonomic assignments at phyla level that do not map directly to microbial functions, as genera within a phylum may have different functions; thus, our ability to make conclusive links between taxa and function is limited.
4. Other important, but less studied, microorganisms that deserve more attention and play critical roles in the BCE carbon cycle and interact with archaea, bacteria, and fungi are protists (unicellular eukaryotes) that convert CO_2_ to organic carbon by the stoichiometry of cellular composition (Worden et al. 2015). In mangrove and seagrass systems, thraustochytrids are increasingly recognised to play important roles in detritus and sediment decomposition due to their production of a wide spectrum of enzymes and should be included in microbial biodiversity assessments (Bongiorni 2011).
5. Better understanding which abiotic drivers (e.g., pH, salinity, hydrology, climate, land use, latitude/longitude) best explain BCE microbial communities. Is pH a major driver, as in terrestrial soils? Is microbial diversity in BCEs linked to climate or anthropogenic disturbances?

## 5 Conclusions

We systematically reviewed 70 years' worth of data spanning 649 publications on archaeal, bacterial, and fungal communities reported from mangrove, saltmarsh, and seagrass ecosystems globally. To our knowledge, this study represents one of the few attempts to comprehensively synthesise published microbial data from BCEs. The main conclusions are:

1. Microbial communities in BCEs are receiving increasing attention, with the number of publications averaging 70 publications in 2020, which constituted 20% of all BCE related papers in that year.
2. In terms of BCEs and their microbial communities, mangroves receive the most attention (66% of publications), followed by saltmarshes (25%), and seagrasses (6%); while 3% of studies included one or more blue carbon ecosystems.
3. Across all studies, bacterial communities were assessed in ~76% of studies, suggesting a higher focus on bacterial communities in BCEs.
4. Compared to bacterial communities, our understanding of archaeal and fungal communities in BCEs, especially in seagrass communities, is scant.
5. We found 80 distinct bacterial functions in mangrove studies, 39 in saltmarsh studies, and 20 in seagrass studies. Archaeal functional diversity was seldom the primary focus of the reviewed studies, with only 0%–10% assessing richness, taxa, or function. Fungal functional diversity was most frequently reported in mangroves, with 16 unique functions, and in saltmarshes, with four unique functions. Overall, despite their crucial role in plant growth and decomposition, our knowledge of soil fungal communities in BCEs remains limited.
6. Current research methods and the use of high‐throughput sequencing are adequate methods to identify BCE microbial communities. However, to better understand microbial contributions to carbon storage, we need to assess their metabolic pathways and how these respond to changes in the abiotic environment and anthropogenic disturbances (Macreadie et al. 2025).
7. Moving away from just taxonomic descriptions of BCE microbial communities to build the profile of functional microbial diversity should be the focus of future BCE microbiome research. For example, complementing amplicon‐based metagenomics (marker‐gene sequencing) with other ‘omics approaches such as whole genome sequencing, which would capture rare and abundant species and enable functional profiling; metatranscriptomics would allow building metabolic functions and metaproteomics would allow gaining direct insights into functional activity.

This review has provided a comprehensive overview of global trends in microbial research, highlighted key knowledge gaps and outlined future directions for microbial research in BCEs.

## Author Contributions

C.B., P.I.M. and S.M.T.‐T. concept, C.B. and P.W. data analysis, C.B. first draft of manuscript; all authors reviewed and approved the draft.

## Conflicts of Interest

The authors declare no conflicts of interest.

## Supporting information


**Figure S1:** Number of studies from mangrove (a), saltmarsh (b) and seagrass (c) ecosystems and reported different methods used for archaeal, bacterial or fungal analyses used across 649 studies reviewed in this synthesis.

**Table S1:** List of 649 studies analysed in this synthesis.

**Table S2:** List of studies that have reported putative functional descriptions from 649 reviewed studies.

**Table S3:** List of studies that have reported both, microbial phyla and associated putative functional descriptions from 649 reviewed studies.

**Table S4:** Relative abundance of reported phyla in BCEs.

## Data Availability

The data that supports the findings of this study are available in the Supporting Information of this article.
