# Supplementary material for: Global Review of Blue Carbon Ecosystem Microbial Communities
Source: Environ Microbiol. 2025 Aug 16;27(8):e70168. doi: 10.1111/1462-2920.70168 (PMC12357167; doi:10.1111/1462-2920.70168)

Supplementary Figure 1: Number of studies from mangrove (a), saltmarsh (b) and seagrass (c) ecosystems and reported different methods used for archaeal, bacterial or fungal analyses used across 649 studies reviewed in this synthesis.


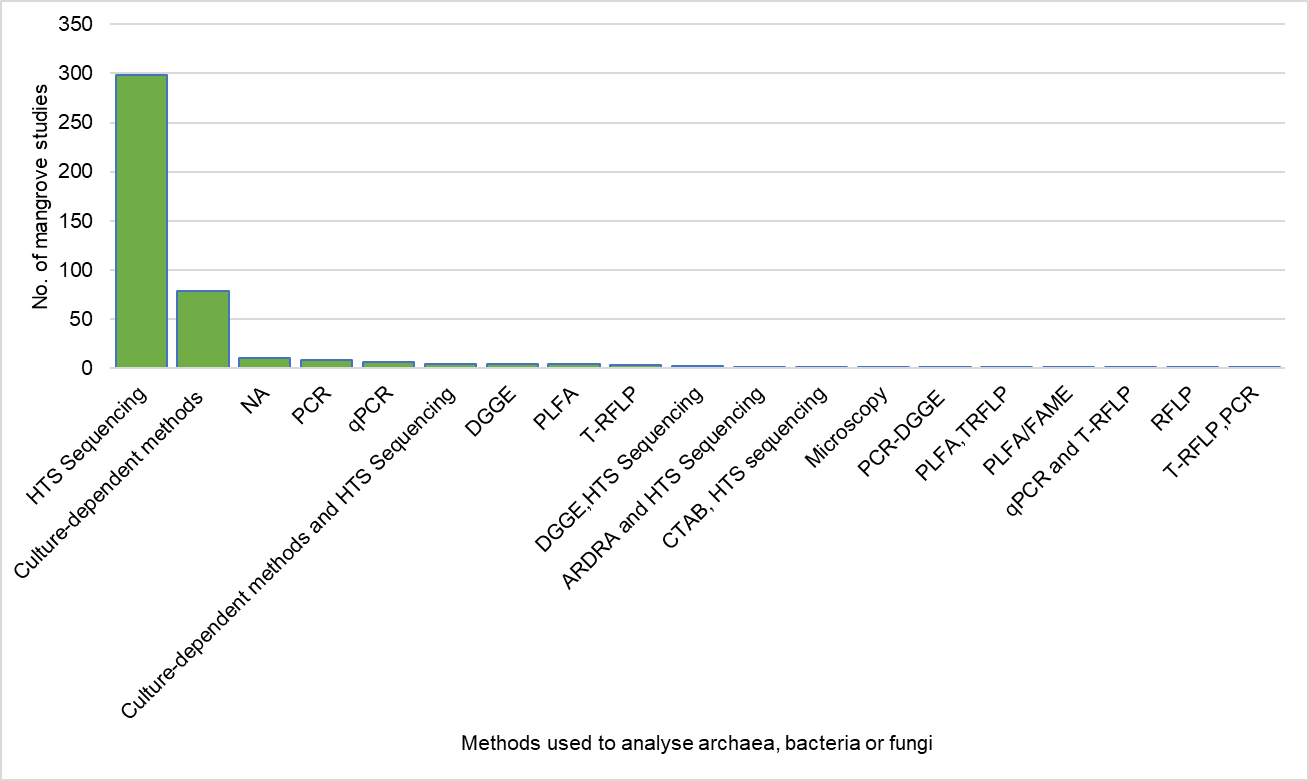


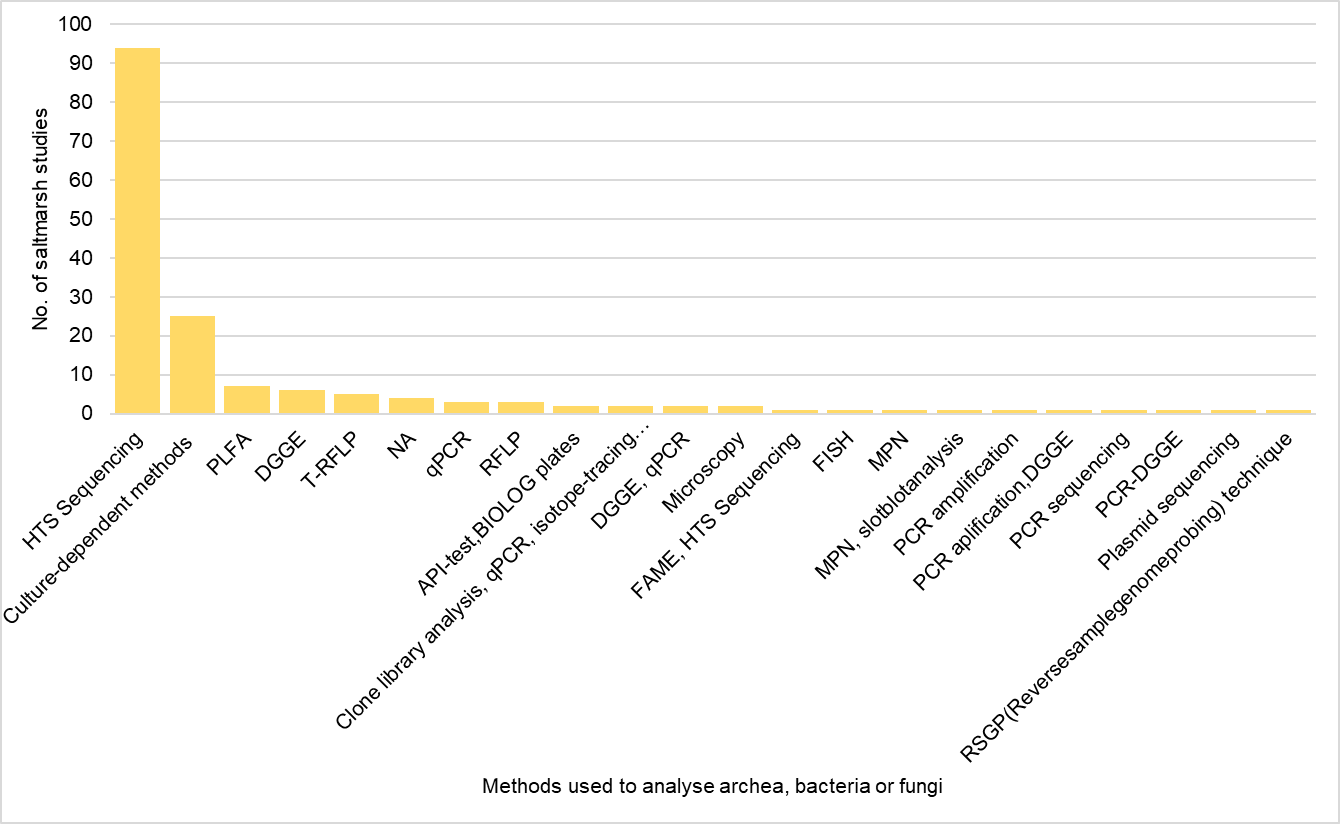


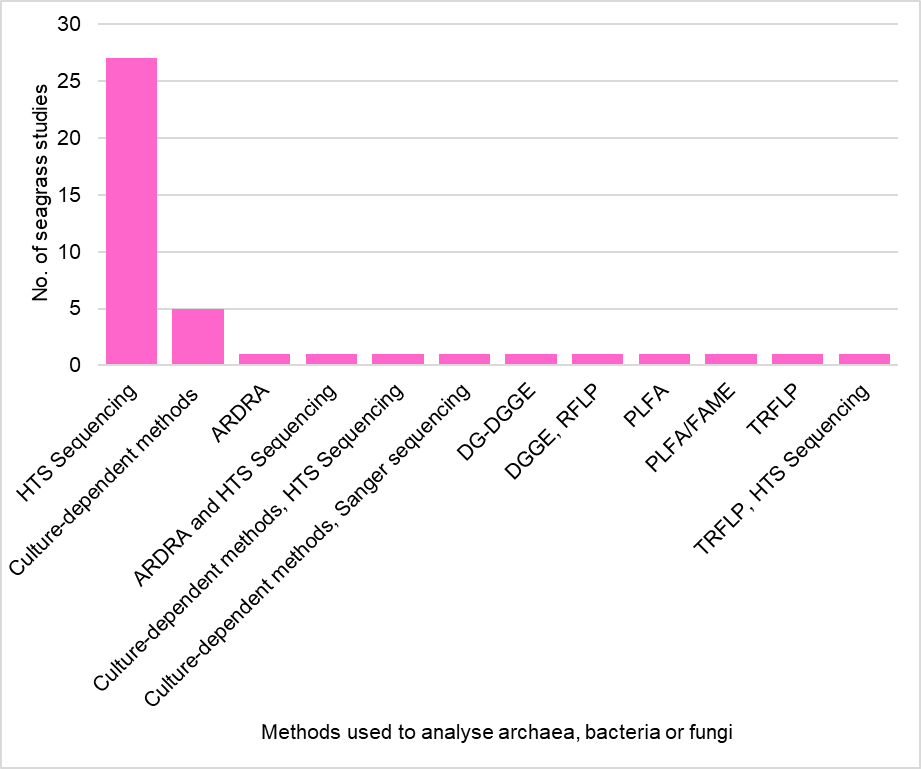

Supplement: Supplementary file 1 — Figure S1: Number of studies from mangrove (a), saltmarsh (b) and seagrass (c) ecosystems and reported different methods used for archaeal, bacterial or fungal analyses used across 649 studies reviewed in this synthesis. [file EMI-27-e70168-s004.docx]
